# Coloarticular Fistula as a Long-Term Sequela of the Hartmann Procedure

**DOI:** 10.7759/cureus.64026

**Published:** 2024-07-07

**Authors:** Jeevan J Murthy, Jake Engel, David DeFazio, Keri Mayers

**Affiliations:** 1 Department of Surgery, West Virginia University School of Medicine, Morgantown, USA

**Keywords:** complication, hip arthropathy, hartmann procedure, diverticulitis, coloarticular fistula

## Abstract

Coloarticular fistula is a rare complication of the Hartmann procedure, reported in the literature only a few times. Risk factors may include underlying colonic malignancy and other proinflammatory conditions. Herein, we describe the case of a 78-year-old female with a recent history of total hip replacement, misdiagnosed Crohn's disease, and a Hartmann procedure for perforated diverticulitis who developed a psoas abscess. Further investigation, including gastrografin enema and computerized tomography, revealed significant fistulization between her hip prosthesis, rectal stump, adnexa, and cecum. Intervention required extensive interdisciplinary decision-making, and the patient underwent interventional radiology-guided abscess drainage, arthroplasty revision, and exploratory laparotomy.

## Introduction

Diverticular disease affects nearly two-thirds of American patients by the age of 85. The etiology of these conditions stems from increased intraluminal pressure causing herniation of colonic mucosa and submucosa through weak portions of the bowel wall, more commonly in the sigmoid colon. The most common risk factors for diverticulosis are age, high-fiber diet, tobacco use, and underlying hypertension [1]. Although typically asymptomatic, diverticulosis predisposes patients to acute diverticulitis, contributing to approximately 280,000 emergency department visits and an annual health expenditure of 8.9 billion dollars [2]. In many cases, complicated and recurrent diverticulitis prompts segmental resection of the diseased portion, an intervention eponymously known as a Hartmann procedure.

The Hinchey classification is a widely accepted system for distinguishing the four levels of complicated diverticulitis. Hartmann procedure is indicated for grades III and IV: colonic perforation with purulent or fecal peritonitis, respectively. Postoperative complications include infection, anastomotic leak, parastomal herniation, and fistula formation. Common types of fistula include colovesical, colovaginal, coloenteric, and colouterine, among others [3]. However, coloarticular fistula formation originating from the rectal stump is a rare phenomenon that has not been previously reported. Herein, we describe the case of a female patient who had undergone a Hartmann procedure with diverting colostomy. Subsequently, she developed a fistula between her rectal stump, cecum, and right hip prosthesis. An abstract of this case was presented at the 2024 American Society of Colon and Rectal Surgeons Annual Scientific Meeting in Baltimore, MD, from June 1 to 4, 2024.

## Case presentation

Our patient is a 78-year-old female with a past medical history of recurrent diverticulitis, right hip arthropathy, atrial fibrillation, diabetes mellitus, and stage 3 chronic kidney disease. She has no known family history of colonic disease but was previously misdiagnosed with Crohn's disease (CD) before being ruled out via computerized tomography (CT) enterography several years after the initial diagnosis. Four years before our introduction, she underwent a Hartmann procedure with end-colostomy secondary to perforated diverticulitis. This operation was complicated by a rectal stump leak, which was managed for several weeks with drain placement. She also received a right-sided hip replacement one year later.

**Figure 1:**
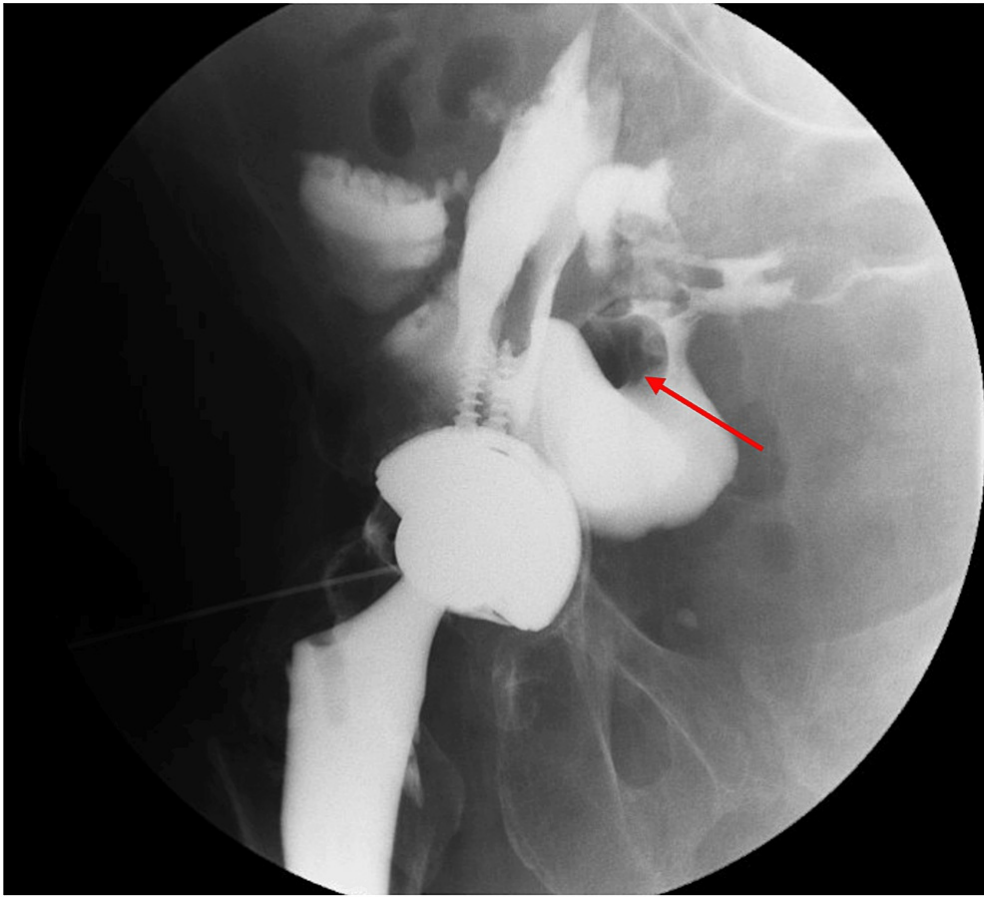
Fistulous tract from the right iliopsoas bursa and hip joint into the lower abdomen communicating with small bowel (red arrow)

**Figure 2:**
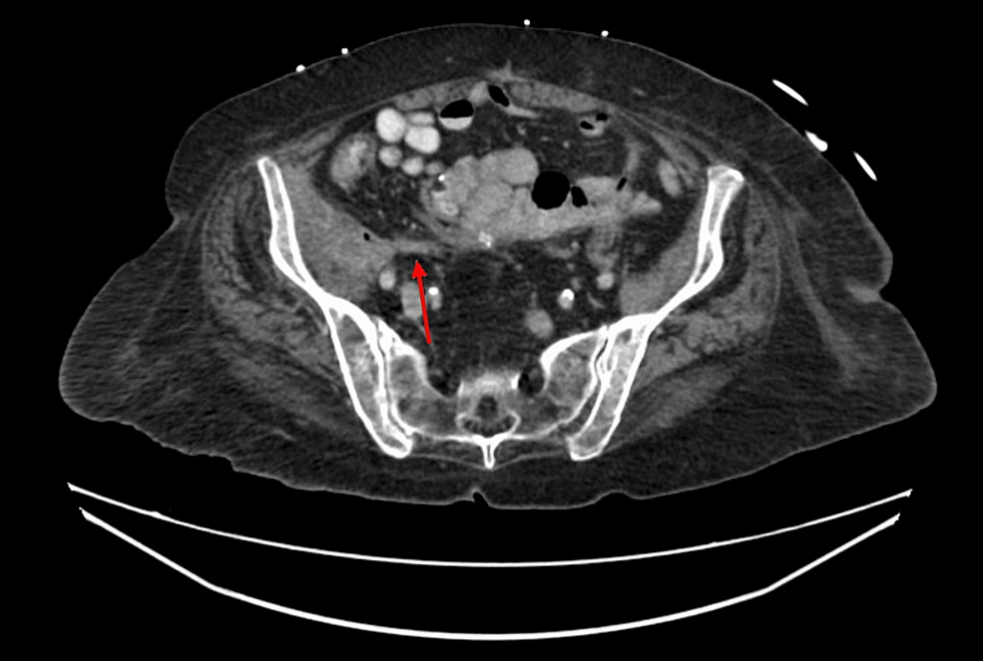
Axial computerized tomography scan with/without intravenous contrast demonstrating complex area containing fluid collection involving the right hip and iliopsoas bursa with extension along the right iliopsoas muscle (red arrow)

**Figure 3:**
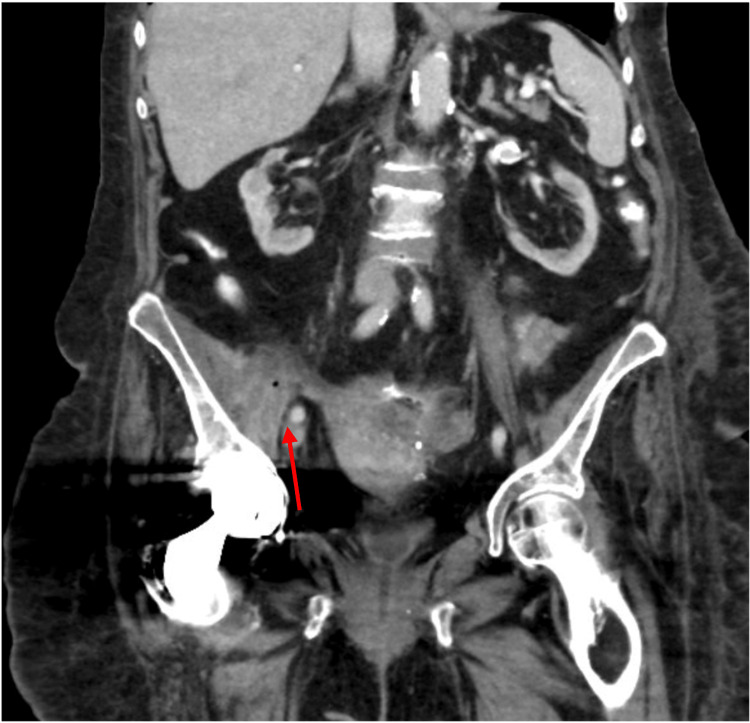
Coronal computerized tomography scan with/without intravenous contrast demonstrating complex area containing fluid collection involving the right hip and iliopsoas bursa with extension along the right iliopsoas muscle (red arrow)

On this occasion, she presented to a tertiary-care colorectal clinic complaining of a recently detected psoas abscess associated with increasing rectal drainage and high volume watery output from her colostomy. Barium enema revealed significant fistulization associated with her right hip prosthesis, cecum, and rectal stump (Figure 1). CT scan of the abdomen and pelvis demonstrated a complicated fistula complex arising from the rectal stump tracking along the retroperitoneum to the right hip and hip arthroplasty (Figures 2, 3).

The patient underwent imaging-guided complex drainage, which yielded a positive fluid culture for *Escherichia coli*, *Streptococcus viridans*, and Actinomyces species. She underwent hip implant resection with placement of an antibiotic-impregnated cement spacer and was discharged on total parenteral nutrition for surgical optimization.

One month later, she was readmitted for exploratory laparotomy. After consenting, anesthesia, patient positioning, and ureteral stenting were completed, an incision was made through her previous laparotomy scar. Small bowel and omental adhesions were lysed sharply using Metzenbaum scissors and electrocautery for over an hour. This permitted better visualization of extensive adherence between loops of jejunum, cecum, rectal staple line, and left peritoneal reflection. Dissection of adhesions and fistulae unavoidably led to several enterotomies, which would eventually require segmental resection and definitive ileocecectomy.

At this point in the procedure, attention was directed toward the pelvic inflammatory process. Direct visualization revealed severe adherence between the uterus, adnexa, and the proximal rectum, along with those previously mentioned. Careful dissection was performed from the pedicle of the inferior mesenteric artery distally, ultimately leading to the division of the diseased rectal stump utilizing a contour stapler. After performing an irrigation bubble test, there was no evidence of air or fluid leak at the new staple line.

Finally, the bowel was inspected from the ligament of Treitz to the colostomy, and hemostasis was confirmed at the side-to-side jejunal and ileocolic anastomoses. An omental flap was fashioned, and 19 Fr Jackson-Pratt drains were placed in the right lower quadrant. Her postoperative course was uneventful, and she was discharged on postoperative day 5. The histopathological specimens from the operation all demonstrated transmural chronic inflammation, hemorrhage, and subserosal fibrosis suggestive of a fistula tract. She continues to follow up with the colorectal clinic and has no evidence of disease recurrence with adequate ostomy function.

## Discussion

Colonic fistula formation following the Hartmann procedure is a known complication. Several retrospective studies report incidences of fistula development ranging from 2% to 6% [4-6]. Enteric fistula tracts more commonly communicate with sites such as the bladder, vagina, and small intestine, but other locations have been reported. In rare cases, a fistula may form after diverting colon surgery, preventing proper healing of the proximal stump, ostomy, or anastomotic ring. Latrille et al. previously described a case of rectal stump fistula to the L4-L5 vertebrae after sigmoid resection [7]. However, our patient differed as her fistula communicated with her hip arthroplasty, which occurred several years after her Hartmann procedure. Numerous cases have previously described GI communication with the hip joint but do not originate from a rectal stump, as seen in our case [8-11]. A meta-analysis from 2002 found that fistula formation following hip arthroplasty was the most common of the GI tract complications [12].

Diversion is a common strategy employed in bowel resection surgery, especially in patients with risk factors such as diverticulitis, malignant colon obstructions, and inflammatory bowel disease (IBD) [13]. This decreases the odds of postoperative perforation and leakage and permits healing of the abdomen. The rectal stump can present its own complications, such as leakage, fistula, diversion proctitis, and stricture. The rectal stump is prone to leak, more commonly a result of inadequate ligation, increasing the risk of pelvic sepsis. A retrospective study looking at risk factors for developing pelvic sepsis after the Hartmann procedure found that 87% of patients with a defective rectal stump staple line developed abscesses [14]. Tactics to mitigate stump leakage include employing indocyanine green fluorescence angiography and intraoperative washout, to name a few [15,16]. Alternatively, the rectal stump has the potential to develop diversion proctitis. Haas and Fox conducted a study in 1990 observing long-term outcomes of rectal pouches after the Hartmann procedure, which found that half of diverticulitis patients and nearly all IBD patients later developed proctitis [17]. Evidence for proper long-term management of the rectal stump after the Hartmann procedure is underreported in literature but represents an important area of investigation [18]. Our patient's abnormal rectal stump fistula development demonstrates the importance of standardized management following the Hartmann procedure.

Our patient was initially misdiagnosed with CD, likely a result of shared nonspecific symptoms and inadequate colon screening. CD is a chronic, incurable transmural IBD of the GI tract, with 70,000 cases diagnosed in the United States annually. Surgical intervention is reserved for the most complex presentations or refractory symptoms such as abscess, stricture, or perforation [19]. On the contrary, diverticulitis is initially treated on an outpatient basis utilizing analgesia and a low-residue diet; however, failure of this course commonly requires inpatient antibiotics [3]. Approximately 15% of these patients experience perforation requiring surgery, as seen in our case [20]. The incorrect diagnosis of CD may have prevented early intervention with the underlying diverticulitis, complicating downstream sequelae. Therefore, tissue diagnosis is of the utmost importance when confirming IBD and ruling out other colonic disorders such as diverticulitis, malignancy, or anorectal disease.

## Conclusions

This case demonstrates the importance of routine postoperative evaluations of complex patients following the Hartmann procedure. Our patient had several risk factors diminishing her healing capacity, including her age, advanced renal disease, and diabetes mellitus. Although fistula formation is a relatively common postoperative complication, it is rare to observe them stemming from a rectal staple line. Combining that with the arthroplasty involvement, this patient experienced unusual sequelae of colonic resection complications. Previous cases have reported rectal arterial, recto-rachidian, and several coloarticular fistulas. However, to our knowledge, this is the first case in the literature demonstrating the formation of a coloarticular fistula originating from the rectal stump. Finally, our patient's misdiagnosis of CD may have prevented timely addressing of the severe diverticular disease. What may have seemed like a typical clinical presentation of CD ended up being a severe case of recurrent diverticulitis. Therefore, clinical judgment should be withheld until it illustrates the importance of ensuring proper clinical, radiological, and pathological diagnoses of the colonic disorder.

## References

[1] Violi A, Cambiè G, Miraglia C (2018). Epidemiology and risk factors for diverticular disease. Acta Biomed.

[2] Peery AF, Crockett SD, Murphy CC (2022). Burden and cost of gastrointestinal, liver, and pancreatic diseases in the United States: update 2021. Gastroenterology.

[3] Ferzoco LB, Raptopoulos V, Silen W (1998). Acute diverticulitis. N Engl J Med.

[4] Samhouri F, Grodsinsky C (1979). The morbidity and mortality of colostomy closure. Dis Colon Rectum.

[5] Desai DC, Brennan EJ Jr, Reilly JF, Smink RD Jr (1998). The utility of the Hartmann procedure. Am J Surg.

[6] Christou N, Rivaille T, Maulat C (2020). Identification of risk factors for morbidity and mortality after Hartmann's reversal surgery - a retrospective study from two French centers. Sci Rep.

[7] Latrille A, Chalumeau C, Fernoux P (2021). Spondylodiscitis on recto-rachidian fistula after non-restored sigmoid resection. J Visc Surg.

[8] Bach CM, Nogler M, Wimmer C, Stoeckel B, Ogon M (2001). Fistula between a total hip arthroplasty and the rectum: a case report. Clin Orthop Relat Res.

[9] Levin JS, Rodriguez AA, Luong K (1997). Fistula between the hip and the sigmoid colon after total hip arthroplasty. A case report. J Bone Joint Surg Am.

[10] Civan O, Ürgüden M (2022). Coloarticular fistula following hip arthroplasty: a report of two cases. Jt Dis Relat Surg.

[11] Long SS, Tawa NE, Ayres DK, Abdeen A, Wu JS (2011). Coloarticular fistula: a rare complication of revision total hip arthroplasty. Radiol Case Rep.

[12] Bach CM, Steingruber IE, Ogon M, Maurer H, Nogler M, Wimmer C (2002). Intrapelvic complications after total hip arthroplasty failure. Am J Surg.

[13] Plasencia A, Bahna H (2019). Diverting ostomy: for whom, when, what, where, and why. Clin Colon Rectal Surg.

[14] Tøttrup A, Frost L (2005). Pelvic sepsis after extended Hartmann's procedure. Dis Colon Rectum.

[15] Iwamoto H, Matsuda K, Hayami S (2020). Quantitative indocyanine green fluorescence imaging used to predict anastomotic leakage focused on rectal stump during laparoscopic anterior resection. J Laparoendosc Adv Surg Tech A.

[16] Schein M, Kopelman D, Nitecki S, Hashmonai M (1993). Management of the leaking rectal stump after Hartmann's procedure. Am J Surg.

[17] Haas PA, Fox TA Jr (1990). The fate of the forgotten rectal pouch after Hartmann's procedure without reconstruction. Am J Surg.

[18] Johnston S, De Lacavalerie P (2020). Management of rectal stump leak following emergency Hartmann's procedure. J Coloproctol.

[19] Lichtenstein GR, Loftus EV, Isaacs KL, Regueiro MD, Gerson LB, Sands BE (2018). ACG clinical guideline: management of Crohn's disease in adults. Am J Gastroenterol.

[20] Lock JF, Galata C, Reißfelder C, Ritz JP, Schiedeck T, Germer CT (2020). The indications for and timing of surgery for diverticular disease. Dtsch Arztebl Int.

